# A proteogenomic analysis of *Shigella flexneri *using 2D LC-MALDI TOF/TOF

**DOI:** 10.1186/1471-2164-12-528

**Published:** 2011-10-28

**Authors:** Lina Zhao, Liguo Liu, Wenchuan Leng, Candong Wei, Qi Jin

**Affiliations:** 1 State Key Laboratory for Molecular Virology and Genetic Engineering, Institute of Pathogen Biology, Chinese Academy of Medical Sciences & Peking Union Medical College, Beijing, PR China; 2 Department of Biological Engineering, College of Life Sciences, Hebei United University, Tangshan City, Hebei Province, P.R. China

## Abstract

**Background:**

New strategies for high-throughput sequencing are constantly appearing, leading to a great increase in the number of completely sequenced genomes. Unfortunately, computational genome annotation is out of step with this progress. Thus, the accurate annotation of these genomes has become a bottleneck of knowledge acquisition.

**Results:**

We exploited a proteogenomic approach to improve conventional genome annotation by integrating proteomic data with genomic information. Using *Shigella flexneri *2a as a model, we identified total 823 proteins, including 187 hypothetical proteins. Among them, three annotated ORFs were extended upstream through comprehensive analysis against an in-house N-terminal extension database. Two genes, which could not be translated to their full length because of stop codon 'mutations' induced by genome sequencing errors, were revised and annotated as fully functional genes. Above all, seven new ORFs were discovered, which were not predicted in *S. flexneri *2a str.301 by any other annotation approaches. The transcripts of four novel ORFs were confirmed by RT-PCR assay. Additionally, most of these novel ORFs were overlapping genes, some even nested within the coding region of other known genes.

**Conclusions:**

Our findings demonstrate that current *Shigella *genome annotation methods are not perfect and need to be improved. Apart from the validation of predicted genes at the protein level, the additional features of proteogenomic tools include revision of annotation errors and discovery of novel ORFs. The complementary dataset could provide more targets for those interested in *Shigella *to perform functional studies.

## Background

New sequencing strategies are constantly under development and are currently able to process a large number of samples with great efficiency in a short period of time. However, accurate annotation of the resulting sequenced genomes has become the bottleneck of knowledge acquisition. Conventionally, most genome sequences are annotated with multiple gene prediction algorithms such as GLIMMER, CRITICA, and GeneMark, or by manual assignment based on BLAST search results [1,2]. Gene density is sufficiently high in prokaryotes, such that coding sequences (CDSs) frequently overlap. Moreover, exon-intron structures present in eukaryotic genomes also make computational annotation difficult. These annotations are rarely experimentally validated, though *in silico *annotation methods could be executed with both high speed and good coverage [3]. The predicted genes exhibit frequent errors, particularly in false recognition of alternative start codons, underestimate of short CDSs, misannotation of pseudogenes, and confusion over overlapping genes. Previous studies have demonstrated that error rates in the definition of translation start sites (TSSs) varied from 10% to 40% in some bacterial and archaeal genomes, according to different computational methods used [4,5]. Likewise, after analysis of overlaps larger than 60 bp among 338 prokaryotic genomes, it was found that the annotation of most previously identified genes was incorrect [6]. In these cases, computational methods were unable to recognize mutations induced by sequencing errors, such as frame-shifts and stop codon mutations. As such, there is a great need for further experimental validation or complementary annotation approaches for conventional genome annotation.

Currently, mass spectrometry (MS)-based proteomic methods are used to address difficulties in gene annotation. Unambiguous identification of proteins by MS is more explicit and confident than that from genomic sequence data alone. The high-throughput nature of shotgun proteomics makes this technology cost-effective and readily reliable to the automated genome annotation process [7,8]. Integrating proteomic information into the genome annotation process, termed proteogenomics [9], directly maps tandem mass spectrometry (MS/MS) spectra data against all six possible reading frames from raw genomic DNA sequences, i.e., experimental proteomic data can be fed back to the genome to aid in the validation of predicted protein-coding genes, potentially avoiding any biases in the computer algorithm. Proteogenomics analysis have already been applied to a number of sequenced prokaryotes and eukaryotes such as the *Mycoplasma pneumonia *[7], *Mycobacterium lepra *[10], *Shewanella oneldensis *[11], *Mycoplasma mobile *[12], *Toxplasma gondii *[13], *Arabidopsis thaliana *[14], human [15] and so on. As a complementary annotation approach, proteomic methods are important for improving the quality of genome annotation, especially for correction of start codon errors by the analysis of a new framework and sequencing of N-terminally acetylated peptides [16-18] and discovery of novel genes missed in the computational genome annotation process [19-23]. Although proteogenomics has made great progress in recent past years, it still highly depends on the results of MS identification, which has its inherent drawbacks, including over representation of highly expressed proteins/peptides and incomplete sampling. Moreover, the sensitivity and throughput of mass spectrometers are also important factors to maximize the benefits of proteogenomic approaches.

*Shigella flexneri *is the primary causative agent of endemic shigellosis in developing countries [24]. Its genome shares a large proportion of chromosomal genes with the model organism *E. coli*. Since 2002, the genomes of four representative strains of species in the family *Shigella *spp. have been sequenced [25,26]. As such, it is an attractive target for proteogenomic annotation. In this study, we applied high-throughput shotgun proteomic technology to explore the comprehensive protein expression profile of *S. flexneri *2a str.301. We completely validated 823 protein products, including hundreds of hypothetical proteins. We also corrected several start sites with the help of our original N-terminal extension database. Furthermore, certain novel open reading frames (ORFs) were confirmed by combining MS analysis and RT-PCR. Our findings suggest that current genome annotations are not yet complete, and that proteogenomic tools have the potential to validate and complement genome wide annotation.

## Results and Discussion

### Validation of annotated ORFs in the *S. flexneri *2a str.301 genome

Raw MS/MS data were used to search a database containing all six possible reading frames of the entire *S. flexneri *2a str.301 genome, using Mascot version 2.2. Applying the filtering criteria described in the Methods section, 823 ORFs from all experiments were unambiguously assigned, of which 811 were previously annotated in the *S. flexneri *2a str.301 genome database from NCBI. (Additional file 1, Table S1). On average, between 2 or 3 peptides were used to identify each ORF, and the amino acid sequence coverage for the detected ORFs averaged 13%. The distribution patterns of pI, Mr, and grand average of hydropathicity (GRAVY) of the identified proteins were similar to those of all *S. flexneri *2a str.301 annotated proteins (Additional file 2, Figure S1-A, B, C). For example, the pI patterns of the identified proteins had the characteristic bimodal distribution, which was previously observed for bacterial and archaeal genomes [27]. Moreover, these proteins (20 of 22 groups in clusters of orthologous groups of proteins, COGs) were involved in nearly all major biological processes (Additional file 2, Figure S2-A, B). Hypothetical proteins were likely to have been annotated incorrectly because of the lack of experimental evidence, and required further experimental validation. In our study, 187 hypothetical or putative ORFs were validated at the protein level, representing 10% of the 1944 predicted hypothetical proteins of *S. flexneri *2a str.301. This was below the average detection rate of all other annotated proteins. Thus, these data suggested that a certain proportion of the hypothetical protein products do not exist in the organism, and represent misannotation of the corresponding genomic region [7,17]. The rest of the peptides that were detected with MS but did not match any annotated protein, are analyzed in detail below. A complete list of identified peptides and their quality scores are given in Additional file 1, Table S1.

### Correction of gene annotation errors

#### Correction of start codon errors

Traditionally, it has been difficult to correctly identify the TSS within a given sequence. For example, a previous study of 143 annotated prokaryotic genomes showed that approximately 60% of the genes might have incorrectly-assigned TSSs [2]. While accurate prediction of TSSs is critical for defining protein sequences, as well as intergenic regions that might contain transcriptional regulatory elements [16]. TSSs were usually verified by N-terminal sequencing analysis. This method was often technically demanding and was not amenable to the majority of proteins with 'blocked', and therefore inaccessible, N-termini [28]. To amend the approximate location of TSSs in these sequences, we developed a proteomic strategy that is simpler than N-terminal sequencing and is also capable of high-throughput analysis, as it is possible that wrongly assigned start sites could be validated and corrected in a single experiment using this method.

All MS-derived peptides were screened against both the *S. flexneri *2a str.301 protein database (downloaded from NCBI) and the customized N-terminal extension database (see Methods section). Peptide hits using the latter indicated that the 5' end of the corresponding gene should be expanded. As a result, three genes (*yhdp*, *yebj*, and *smpA*) were identified as having true start codons upstream of their current start codons (Table 1; Additional file 2, Figure S3). In addition, by performing a BLASTP search against GenBank, the N-terminus extended proteins other than the original proteins shared higher similarities with their homologs in other bacteria (data not shown). Moreover, we successfully designed primers based on the N-terminal extension region for RT-PCR experiments to confirm the existence of the three extended genes (Additional file 2, Figure S4), suggesting that the N-terminal extensions inferred by our method were reliable. The initial codons of all three genes were corrected and updated in GenBank entries based on our new evidence. This original strategy of combining both N-terminal proteomic analysis and transcriptional verification represents an effective and promising means for experimental identification of TSSs. We expect that this strategy can be applied to other organisms.

**Table 1:** N-terminal extension of three genes

Gene	Tag	Predicted start site	Updated state site	Old start codon	New start codon	Peptides matching N-terminal extension database	Peptide score
*yhdP*	BIO47422	3382990	3383830	GTG	GTG	DLTFWQLR	52
*yebJ*	BIO00465	1434566	1433987	GTG	ATG	IGIFQDLVDR	55
						VDLDGNPCGELDEQHVEHAR	101
*smpA*	BIO00925	2752334	2752145	ATG	ATG	VVYRPDINQGNYLTANDVSK	85

#### Correction of sequencing errors

**Figure 1:**
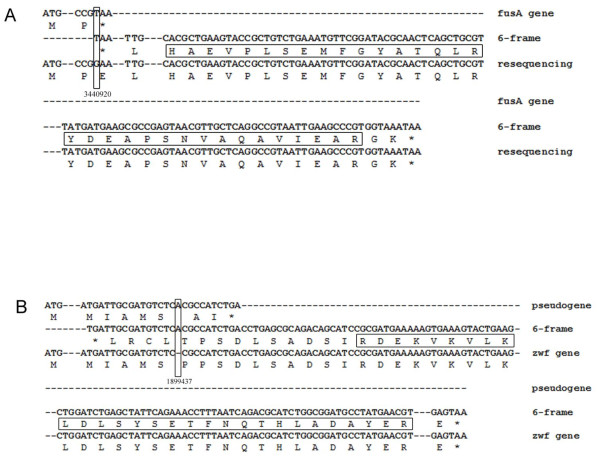
**Examples of sequencing errors identified by proteogenomic analysis**. (A) The nucleotide and corresponding amino acid sequences of the *fusA *gene. The 'G' at genome position 3, 440, 920 was previously erroneously recognized as 'T', resulting in a stop codon mutation. (B) The nucleotide and corresponding amino acid sequences of the *zwf *gene and its pseudogene. An extra 'A' at genome position 1, 899, 437 resulted in a frameshift that caused a premature termination mutation. These two sequencing errors were corrected in GenBank entries on our request. Unambiguously assigned peptides and sequencing error bases are boxed. *, stop codon.

Although genome sequencing technologies have made great progress in the last 10 years, none of these next-generation sequencing methods are 100% accurate. There are usually a few wrong bases in an otherwise accurate genome. With the aid of proteogenomic tools, we could uncover genes that contained certain avoidable sequencing errors, which usually led to erroneous annotations. For example, we found an ORF (*fusA*) in *S. flexneri 2a *str.301, which was 240 bp shorter at the 3' end than its homologs in other *Shigella *genomes. However, our MS/MS data identified peptides matching the missing part of *fusA *(BIO01150) in *S. flexneri *2a str.301 (Figure 1A). To test if a stop codon mutation resulted from a sequencing error, we re-sequenced the coding region of *fusA *and found that the guanine at genome position 3, 440, 920 was previously recognized as thymine, because of a mistake in the initial genome sequencing project. This sequencing error led to a transformation from GAA (coding Glu) to the premature termination codon TAA (Figure 1A). As a result, the 3' end of the *fusA *gene annotated in *S. flexneri *2a str.301 should be extended from 3, 440, 918 to 3, 440, 678. Importantly, this gene is now seen to encode a full-length protein product.

Bacterial pseudogenes were originally considered to be infrequent. Despite having DNA sequences similar to those of known genes, pseudogenes were regarded as disabled copies of functional genes [29]. Nonetheless, we detected two peptide segments matching the protein product of the *zwf *(BIO80170) pseudogene, which was orthologous to *E. coli *K12 glucose-6-phosphate dehydrogenase (Figure 1B). Was the *zwf *gene in *S. flexneri *2a str.301 a true pseudogene? Further re-sequencing of the regional genomic sequence revealed that there was an extra adenine insertion into the coding region of *zwf *in the original *S. flexneri *2a str.301 genome sequence, which resulted in frame-shift introducing a premature stop codon (Figure 1B). As such, based on our proteogenomic finds, the *zwf *pseudogene in *S. flexneri *2a str.301 was revised to encode a functional full-length product. The *yraJ *gene, which encodes an outer membrane usher protein in other enterobacteria, was disrupted by an IS2 insertion sequence in *S. flexneri *2a str.301. Using the six-reading-frame database search, we identified this pseudogene's premature protein product (BIO11778). Its transcript was also detected by RT-PCR (Additional file 2, Figure S5). Previous studies revealed that the intact usher protein assembled in the OM as a dimeric secretion complex [30]. From an evolutionary standpoint, it has been considered that transcribed/translated pseudogenes were not necessarily without function. How the premature protein functions remains to be determined.

### Discovery of novel ORFs

The most striking result of our study was the identification of novel ORFs. All assigned ORFs were aligned with the current annotated ORFs of *S. flexneri *2a str.301 using BLASTP, and those that aligned with annotated proteins were discarded. As a result, we detected 7 novel ORFs that were not predicted in *S. flexneri *2a str.301 by any other annotation pipelines (see Table 2). Among these novel ORFs, four ORFs have orthologs in other closely-related organisms, which allowed substantial cross-species validation of the new genes. Significantly, the other three ORFs were completely novel genes that had no homology with other annotated proteins from any species.

**Table 2:** Characteristics of seven novel ORFs

Gene tag	Strand	Length (AA)	**Overlaps**^**a)**^	Annotation in other enterobacteria
BIO01608^b)^	+	80	No	Hypothetical protein
BIO50043^b)^	-	365	Partial (S)	Sulfate/thiosulfate transporter subunit
BIO07235^b)^	+	25	Partial (S)	None
BIO43803^b)^	-	496	Partial (C)	Hypothetical protein
BIO68373	-	59	Nested (C)	Conserved hypothetical protein
BIO58539	-	86	Nested (S)	None
BIO48527	-	36	Nested (S)	None

a) No, ORFs not overlapping other genes; Partial (C), ORFs partially overlapping known genes on the complementary strand; Partial (S), ORFs partially overlapping known genes on the same strand; Nested (C), ORFs completely contained within known genes on the complementary strand; Nested (S), ORFs completely contained within known genes on the same strand, but in a different frame.

b) The transcripts of novel ORFs were confirmed by RT-PCR assay.

**Figure 2:**
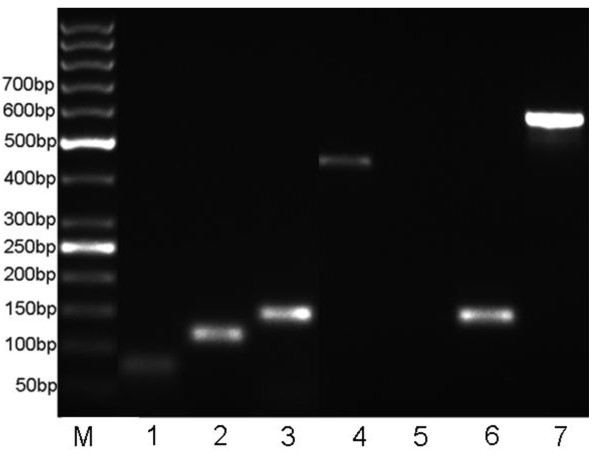
**Validating MS data using RT-PCR**. RNA fragments of the expected sizes were observed, indicating that these un-annotated genes are transcribed. Figure shows the RT-PCR verification results for six novel genes. Amplified PCR products were electrophoresed on a 2.5% agarose gel and visualized by ethidium bromide staining. BIO07235, BIO01608, BIO43803, and BIO50043 were amplified and loaded in lanes 1-4, respectively; a negative control (noncoding DNA sequence) was loaded in lane 5 (cDNA as template) and lane 6 (genomic DNA as template); Lane 7, positive control (housekeeping gene, ipaD); Lane M, GeneRuler™ 50 bp DNA Ladder (Fermentas GmbH, Germany).

We focused on the seven novel genes to further investigate why they escaped computational prediction. First, these novel ORFs were relatively short. To our knowledge, short CDSs (especially less than 150 nucleotides) are among the most difficult genomic features to predict and are often missed during the annotation process due to conservative calls [8]. On the other hand, most of the identified novel ORFs were partially or entirely overlapped by annotated longer ORFs (Table 2; Additional file 2, Figure S6). For gene prediction software, the percentage of missing genes is strongly correlated with the frequency of gene overlaps. In Glimmer, the maximum overlap length is set to 30 bp by default [31]. Generally, the relatively longer ORF rather than its overlapping genes is likely to be retained. Unfortunately, those omitted overlapping genes might be true genes [23]. As Figure S6 shown, generally there were four patterns for the relative location of overlapping gene pairs. Of the seven novel ORFs, one ORF (BIO01608) had no overlap with known genes and other three ORFs (BIO50043, BIO07235, BIO43803) respectively belonged to pattern I or pattern II, whose transcripts were easy to be verified by RT-PCR assays. The results showed that the transcripts of four ORFs were specifically detected (Figure 2), and additional sequencing of these PCR products confirmed their identity. The rest three ORFs (BIO58539, BIO48527, BIO68373) were entirely contained within the coding region of certain longer known genes (Pattern III or Pattern IV), referred to as "nested" genes. Although nested genes are quite rare in prokaryotic genomes, this kind of gene arrangement is beginning to be recognized, such as *setBA */*pic *in *S. flexneri *2a [32,33], *ins5B */*ins5A *and *hgtA/yaa*W in *E. col*i [34,35], *and Pfl01_0939/cosA in P. Fluorescens *[36]. The existence of nested genes increases the organizational complexity of the genome structure, so it is not practical to investigate all gene arrangements during conventional genome annotation. As such, proteogenomic methods offer a promising avenue toward the experimental validation of nested genes at the protein level [37].

Short CDSs remain largely unknown, even though small peptides encoded by short genes are involved in diverse functions, such as secretion, stress responses, metabolism, and gene regulation in bacteria [38,39]. We also examined the gene structure of each of the seven novel genes. In our study, there were no identifiable functional domains in the seven novel ORFs, with the exception of BIO01608 and BIO50043. BIO01608 contains an YmgB superfamily conserved domain, which is involved in biofilm development and stability. BIO50043 contains an ABC_CysA_sulfate_importer conserved domain, which is involved in sulfate import, and whose ortholog in E. coli is annotated as a sulfate/thiosulfate transporter subunit. Overlapping gene pairs were conserved among organisms for specific genes and functions. In addition, it was generally assumed that their expressions were correlated with host genes, which would reduce the need for more complex regulatory pathways and thus the regulation of gene expression would be more effective [1,40]. For example, of the *setBA */*pic *nested gene pair in *S. flexneri *2a, the *pic *gene encodes mucinase, which is involved in mucosal colonization, and *set1A *and *set1B *encode the two subunits of the ShET1 enterotoxin. The two partners were likely to be expressed reciprocally and function cooperatively [41], which aided our further investigation of the function of nested gene pair. Exploring these novel nested genes' biological functions and their coordination with host genes are under investigation.

## Conclusions

In our study, the detection of annotation errors, such as incorrect start sites assignment, sequencing errors, and wrongly annotated pseudogenes, would prevent misannotation from being multiplied in future versions of the *S. flexneri *2a str.301 genome. The findings of novel ORFs would also provide a new clue to conduct functional research. Moreover, some of the novel ORFs were identified as overlapping genes, which increases our understanding of the complexity of the genome structure and reveals the underestimation of such gene arrangements. This updated dataset would be very helpful for those interested in this pathogen to unearth certain information previously omitted. With the rapid development of proteomic technology, all sequence-based genome projects could be supplemented by the proteogenomic analysis.

## Methods

### Strain and culture conditions

Frozen *S. flexneri *2a, str.301 (kindly provided by the ICDC, China CDC) cell stocks were streaked onto tryptic soy agar containing 0.01% Congo red. An individual red colony was subsequently transferred into tryptic soy broth (TSB) and grown overnight at 37°C with rotary shaking at 200 rpm. The overnight culture was diluted 1:50 in fresh TSB and incubated under the same conditions until OD_600 _= 0.6-1.0. Cells were harvested by 8 min centrifugation at 2, 500 × *g *at 4°C and then washed twice ice-cold 50 mM Tris-HCl, pH 7.3. The pelleted cells were frozen at -20°C until required.

### Sample pre-fractionation procedures

Bacterial cells were resuspended in 100 mM Tris-HCl buffer (pH 8.5), containing 7 M Urea, 2 M Thiourea, a protease inhibitor cocktail tablet (Roche Diagnostics, Germany), and Benzonase Nuclease (25 U/ml, Sigma, USA), and then ruptured by ultrasonication. The unbroken cells were removed by centrifugation at 4, 000 × *g *for 10 min at 4°C. The supernatant was diluted with ice cold 100 mM Na_2_CO_3 _(pH 11.5) to a final pH 11 and stirred slowly on ice for 1 h. The supernatant was further collected by ultracentrifugation in a Beckman SW 40Ti rotor at an average of 150, 000 × g for 1 h at 4°C. The supernatant was analyzed for cytosolic protein components. The membrane pellet was resuspended and washed twice in ice-cold 100 mM Na_2_CO_3 _(pH 11.5) at 4°C. Finally, the washed membrane sheets were pelleted by ultracentrifugation at an average of 150, 000 × *g *for 45 min and resuspended in 100 mM NH_4_HCO_3 _containing 7 M Urea and 2 M Thiourea [42]. Cytosolic and membrane fractions were measured for protein content using a bicinchoninic acid (BCA) assay. Both of fraction samples were analyzed in parallel and replicated three times.

### In-solution digest

Cytosolic and membrane fractions were reduced in the presence of 10 mM DTT at 37°C for 45 min, and then alkylated in the presence of 50 mM iodacetamide at room temperature in the dark for 30 min. The reaction products were diluted to 1 M urea and digested with trypsin (1:50 w/w, modified sequencing grade, Promega, USA) overnight at 37°C. Peptides were desalted using an Oasis HLB extraction cartridge (Waters, USA). All peptide fractions were concentrated with a Speed-vac centrifuge (Eppendorf, Germany) and resolubilized in 0.1% TFA for the following two-dimensional liquid chromatography matrix-assisted laser desorption/ionization (2D LC-MALDI) analysis.

### 2D LC-MALDI analysis

Digested peptide mixtures were separated using the Ultimate 3000 HPLC system (Dionex-LC-Packings, USA) coupled with a PROTEINEER fc LC-MALDI fraction collector (Bruker, Germany). The HPLC system consisted of a strong cation exchange (SCX) column (300 μm id POROS 10S Column, Dionex) and a C18 reverse-phase microcapillary column (PepMap100 C18, 300 μm, 100Å, Dionex). The flow rate through the column was 2 μl/min. The solutions used were as follows: 0.05% TFA in water (buffer A), 0.04% TFA in 80% ACN (buffer B). A sample of the desired peptides digest was loaded onto the SCX column. NaCl of different concentration at 0.5, 1, 2, 3, 5, 10, 25, 50, 100, 200, 500, 1000 mM was used to displace peptide fractions from the SCX column onto the RP column, respectively. Each case was synchronized with a 90 min RP gradient. Gradient conditions: isocratic pre-run at 4% B, 0-5 min; linear gradient 4-65% B, 5-65 min; 65-100% B, 65-70 min, column wash at 100% B, 70-75 min; re-equilibrate the column at 4% B, 75-90 min. Online MALDI spotting of LC fractions was carried as follows: number of fractions, 384 (covering the gradient 4-65% B); MALDI target, Pre-spotted disposable AnchorChip PAC 384 HCCA target (Bruker, Germany); fraction width, 15 s (500 nl). MS spectra were automatically acquired on an Ultraflex III MALDI-TOF/TOF mass spectrometer (Bruker Daltonics, Germany) in the positive reflection mode under the control of Compass 1.2 and WARP-LC 1.0 software (Bruker Daltonics, Germany). The parameter settings were: 20 kV accelerating voltage and 23 kV reflecting voltage; MS and MS/MS mass range: *m*/*z *700-4000 and 50-2000, respectively; Detected peptide compounds with a signal-to-noise ratio higher than 10 were subjected to MALDI-time of flight (TOF) MS/MS analysis.

### In-house database construction

We translated the *S. flexneri *2a str.301 genome (downloaded from NCBI) into all six possible reading frames, generating a set of all possible peptides (larger than 15 amino acids) that could be encoded. Sequences for common contaminants from two collections (248 from Max Planck Institute of Biochemistry, 112 from the Global Proteome Machine Organization Common Repository of Adventitious Protein), were merged into one (total 338 unique entries) and appended to the end of the above target database FASTA file. The final database had 90330 entries. To detect potential extended TSSs of the predicted coding sequences, we constructed a specialized N-terminal extension database, using a similar strategy as previously described [16] with some changes. The database took into account all currently annotated CDSs from the *S. flexneri 2a *str.301 genome. The region upstream of each CDS was scanned until an in-frame stop codon was identified. Then, the in-frame codons downstream of this stop codon were scanned for the first location of a start codon (ATG, GTG or TTG). The peptide from the new start codon to the 33rd amino acid residue downstream of original start site was collected into the extension database, except for those CDSs whose start codon was the same as the previous annotation. As a result, 1311 peptides were collected in the customized extension database (Additional file 3, Table S2).

### Data evaluation

MS/MS data were searched using Biotools 3.1 software (Bruker Daltonics, Germany) with MASCOT 2.2 plugin http://www.matrixscience.com against the six reading frame translation of *S. flexneri *2a str.301 genome. All MS/MS spectra were deposited into the PRIDE database [43]http://www.ebi.ac.uk/pride/ and could be downloaded from this URL: http://www.mgc.ac.cn/Resources/mzXML_S.flexneri_WARP-LC.tgz. The following search parameters were applied: max missed cleavage: 1; fixed modification: Carbamidomethylation (C); variable modification: Oxidation (M), Carbamyl (N-term), Deamidated (NQ); precursor ion mass tolerance: ± 50 ppm; fragment mass tolerance: ± 0.6 Da. Decoy searches were performed using the automated 'Decoy' search option from Mascot. In this strategy, Mascot will generate and search a random version of each target database protein. The false discovery rate (FDR) is calculated as follows:

$$\text{FDR}=\text{Decoy hits}\left(\text{FP}\right)∕\text{Target hits }\left(\text{FP}+\text{TP}\right).$$

We tweaked the peptide significance threshold (at most 0.01) to control the FDR value under 1%. Under these criteria, all the proteins with at least one unique peptide identification at *p *< 0.01 were considered likely to be present in the sample. Additionally, total proteins identified by a single peptide and all novel protein identifications could not be accepted unless their corresponding MS/MS spectra passed the manual validation. All spectra used for annotated ORF identifications based on unique peptides (ion score < 45), as well as all those of novel ORFs are shown in Additional file 4.

### RT-PCR

Total RNA of *S. flexneri *2a str.301 was extracted using the SV Total RNA Isolation System Kit (Promega, USA) following the manufacturer's protocol. Total RNA was treated with RQ1 RNase-free DNase (Promega, USA) to remove residual genomic DNA, followed by heat inactivation of the endonuclease. cDNA synthesis was performed from 1 μg of RNA using the SuperScript™ III Reverse Transcriptase (Invitrogen, USA) according to the manufacturer's protocol. PCR was performed using 1 μl of the reverse transcription reaction as a starting material according to standard procedures. PCR cycling parameters were typically 4 min at 94°C; 30 cycles of 30 s at 94°C, 30 s at 55°C, 30 s at 72°C; and a final 10 min extension at 72°C. The RT-PCR assay was run with the housekeeping gene (*ipaD*) as a positive control and a non-coding DNA sequence (from 417, 540 to 417, 690 in the *S. flexneri *2a str.301 genome) as the negative control. Gene-specific primers used to amplify the target genes are listed in Additional file 5, Table S3.

## Competing interests

The authors declare that they have no competing interests.

## Authors' contributions

LZ carried out the proteomic experiment, participated in the sequence alignment, and drafted the manuscript. LL carried out the RT-PCR assay. WL participated in the MS analysis. CW participated in the design of the study, performed the statistical analysis, and helped to draft the manuscript. QJ conceived the study, and participated in its design and coordination. All authors read and approved the final manuscript.

## Supplementary Material

Additional file 1 **Supplementary Table S1, All proteins identified by MS analysis**. This file contains detailed information of all identified proteins in our study. Click here for file

Additional file 2 **Supplementary Figures**. This file contains supplementary Figures S1-6. Figure S1 illustrates the patterns of pI, Mr, and GRAVY value of identified/annotated proteins. Figure S2 illustrates COGs functional categories of identified/annotated proteins. Figure S3 shows the information about N-terminal extension of three genes. Figure S4 shows RT-PCR results of three extended genes. Figure S5 shows RT-PCR results for BIO11778. Figure S6 illustrates patterns for relative location of novel ORFs overlapping known genes. Click here for file

Additional file 3 **Supplementary Table S2, List of candidate N-terminal extension genes**. This file contains a list of genes that are likely to be extended at the N-terminus in the *S. flexner*i 2a str.301 genome. Each entry's information includes locus tag, extension region: genome position of region from N-terminal new start codon to the 33rd amino acid residue downstream of original start site for each extended gene, and the peptide sequence corresponding to extension region. Click here for file

Additional file 4 **Manually validated MS/MS spectra**. This file shows all MS/MS spectra of peptides matching to annotated proteins that had a single peptide hit (ion score < 45) and un-annotated novel proteins. Click here for file

Additional file 5 **Supplementary Table S3**. Table S3 shows a list of primers used in this article. Click here for file
