# Supplementary material for: A proteogenomic analysis of Shigella flexneri using 2D LC-MALDI TOF/TOF
Source: BMC Genomics. 2011 Oct 28;12:528. doi: 10.1186/1471-2164-12-528 (PMC3219829; doi:10.1186/1471-2164-12-528)
Supplement: Additional file 2 — Supplementary Figures. This file contains supplementary Figures S1-6. Figure S1 illustrates the patterns of pI, Mr, and GRAVY value of identified/annotated proteins. Figure S2 illustrates COGs functional categories of identified/annotated proteins. Figure S3 shows the information about N-terminal extension of three genes. Figure S4 shows RT-PCR results of three extended genes. Figure S5 shows RT-PCR results for BIO11778. Figure S6 illustrates patterns for relative location of novel ORFs overlapping known genes. [file 1471-2164-12-528-S2.DOC]

### Additional file 2 – Supplementary Figures

B

A

C

**Figure S1. Patterns of pI,** **Mr, and GRAVY values of identified/predicted proteins.** (A) Comparison of pI between identified proteins and predicted proteins; (B) Comparison of Mr between identified proteins and annotated proteins; (C) GRAVY distribution of identified proteins.

A

B

A

B

**Figure S2****. COGs functional categories of identified/predicted proteins.** (A) COGs functional classification of identified proteins; (B) Comparison of COGs functional classification between identified proteins and predicted proteins.

1. *yhdP* （BIO47422）

… … … … … … …

GTG... … … …

GTG … … … …

Peptide ID I

Sense Primer

… … … … … … …

… … … … … … …

Antisense Primer

Peptide ID II

… … … TGA

-840 （DLTFWQLR） +1 (QSSFNVDYDLHWR)

1. *yebj* （BIO00465）

… … … … … … …

ATG... … … …

Peptide ID I

GTG … … … …

Sense Primer

… … … … … … …

Antisense Primer

Peptide ID II

… … … … … … …

… … … TGA

-579 (IGIFQDLVDR) (VDLDGNPCGELDEQHVEHAR) +1

1. *smpA* （BIO00925）

… … … TAA

… … … … … … …

ATG... … … …

ATG … … … …

Sense Primer

Antisense Primer

Peptide ID I

-189 (VVYRPDINQGNYLTANDVSK) +1

**Figure S3. N-terminal extension of three genes.** This figure showed the information of three extended genes (*yhdP, yebj* and *smpA* **) :** the nucleic acid region indicating the predicted +1 of transcription, the RT-PCR amplified fragment (the fragment between sense primer and antisense primer), the previous and the novel translation initiation codons, and the peptide evidence. The initial codons of all three genes were corrected and updated in GenBank entries based on our new evidence.


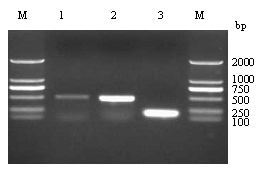


**Figure S4**. **RT-PCR results for three extended genes.** The results indicated that the three annotated ORFs were extended upstream to some extent. M: DL2000; Lane1: *yhdP*; Lane 2: *yebJ*; Lane 3: *smpA*.


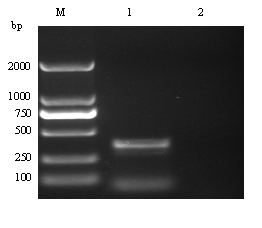


**Figure S5**. **RT-PCR result of BIO11778.** The forward primer was designed to anneal to the template in the region of the start codon (Lane 1) or upstream of the start codon (Lane 2) of the annotated pseudogene *yraJ*. The result confirmed the transcription of the *yraJ* pseudogene.

**A**

Pattern I (Partial S)

Gene 1

Gene 2

Gene 1

Gene 2

Pattern II (Partial C)

Gene 1

Gene 1

Gene 2

Gene 2

Pattern III (Nested S)

Gene 1

Gene 2

Pattern IV (Nested C)

Gene 1

Gene 2

**B**

1. BIO50043 (Pattern I)

*cys W* (450 bp)

BIO50043 (1098 bp)

1. BIO07235 (Pattern I)

*yddM* (363 bp)

BIO07253 (78bp)

1. BIO43803 (Pattern II)

*YjjG* (678 bp)

BIO43803 (1491 bp)

*prfC* (1590 bp)

1. BIO068373 (Pattern IV)

*SF1133* (747 bp)

BIO068373 (180bp)

1. BIO58539 (Pattern III)

BIO58539 (261 bp)

*yjbH* (2097 bp)

1. BIO48527 (Pattern III)

BIO48527 (111 bp)

*yqgB* (147 bp)

**Figure S6. Patterns for relative location of novel ORFs overlapping known genes.** (A) Four arrangements of overlapping gene pairs. Pattern I (Partial S): ORFs partially overlapping known genes on the same strand; Pattern II (Partial C): ORFs partially overlapping known genes on the complementary strand; Pattern III (Nested S): ORFs completely contained within known genes on the same strand, but in a different frame; Pattern IV (Nested C): ORFs completely contained within known genes on the complementary strand. (B) Structure organization of six novel genes and its corresponding host genes in this study.
