# Supplementary material for: A proteogenomic analysis of Shigella flexneri using 2D LC-MALDI TOF/TOF
Source: BMC Genomics. 2011 Oct 28;12:528. doi: 10.1186/1471-2164-12-528 (PMC3219829; doi:10.1186/1471-2164-12-528)
Supplement: Additional file 4 — Manually validated MS/MS spectra. This file shows all MS/MS spectra of peptides matching to annotated proteins that had a single peptide hit (ion score < 45) and un-annotated novel proteins. [file 1471-2164-12-528-S4.DOC]

### Additional file 4 –Manually validated MS/MS spectra

The files present all MS/MS spectra of peptides matching to annotated proteins that had a single peptide hit (ion score<45) and all novel proteins in our study. Each entry is represented by sequence, iron score, validation result and MS/MS spectra of matching peptides.

1. Annotated proteins MS/MS spectra

BIO87809

SLNLSIEAPSGAR 44.9


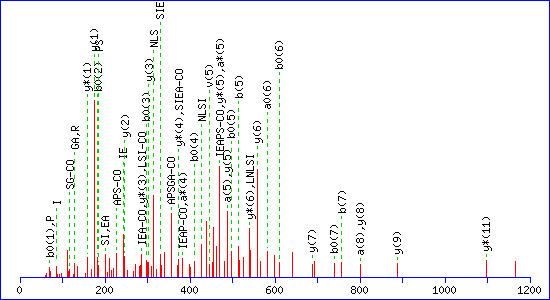


| **#** | **Immon.** | **a** | **a*** | **a^0^** | **b** | **b*** | **b^0^** | **Seq.** | **v** | **y** | **y*** | **y^0^** | **#** |
| --- | --- | --- | --- | --- | --- | --- | --- | --- | --- | --- | --- | --- | --- |
| **1** | 60.0444 | 60.0444 |  | 42.0338 | 88.0393 |  | **70.0287** | **S** |  |  |  |  | **13** |
| **2** | **86.0964** | 173.1285 |  | 155.1179 | **201.1234** |  | **183.1128** | **L** | 1169.5909 | 1227.6692 | 1210.6426 | 1209.6586 | **12** |
| **3** | 87.0553 | 287.1714 | 270.1448 | 269.1608 | **315.1663** | 298.1397 | **297.1557** | **N** | 1055.5480 | 1114.5851 | **1097.5586** | 1096.5745 | **11** |
| **4** | **86.0964** | 400.2554 | **383.2289** | 382.2449 | **428.2504** | 411.2238 | **410.2398** | **L** | 942.4639 | 1000.5422 | 983.5156 | 982.5316 | **10** |
| **5** | 60.0444 | **487.2875** | **470.2609** | 469.2769 | **515.2824** | 498.2558 | **497.2718** | **S** | 855.4319 | **887.4581** | 870.4316 | 869.4476 | **9** |
| **6** | **86.0964** | 600.3715 | 583.3450 | **582.3610** | 628.3665 | 611.3399 | **610.3559** | **I** | 742.3478 | **800.4261** | 783.3995 | 782.4155 | **8** |
| **7** | 102.0550 | 729.4141 | 712.3876 | 711.4036 | **757.4090** | 740.3825 | **739.3985** | **E** | 613.3052 | **687.3420** | 670.3155 | 669.3315 | **7** |
| **8** | 44.0495 | **800.4512** | 783.4247 | 782.4407 | 828.4462 | 811.4196 | 810.4356 | **A** | 542.2681 | **558.2994** | **541.2729** | 540.2889 | **6** |
| **9** | **70.0651** | 897.5040 | 880.4775 | 879.4934 | 925.4989 | 908.4724 | 907.4884 | **P** | **445.2154** | **487.2623** | **470.2358** | 469.2518 | **5** |
| **10** | 60.0444 | 984.5360 | 967.5095 | 966.5255 | 1012.5310 | 995.5044 | 994.5204 | **S** | 358.1833 | 390.2096 | **373.1830** | 372.1990 | **4** |
| **11** | 30.0338 | 1041.5575 | 1024.5310 | 1023.5469 | 1069.5524 | 1052.5259 | 1051.5418 | **G** |  | **303.1775** | **286.1510** |  | **3** |
| **12** | 44.0495 | 1112.5946 | 1095.5681 | 1094.5840 | 1140.5895 | 1123.5630 | 1122.5790 | **A** | 230.1248 | **246.1561** | 229.1295 |  | **2** |
| **13** | **129.1135** |  |  |  |  |  |  | **R** | 74.0237 | **175.1190** | **158.0924** |  | **1** |

BIO77903

VIDHYENPR 43


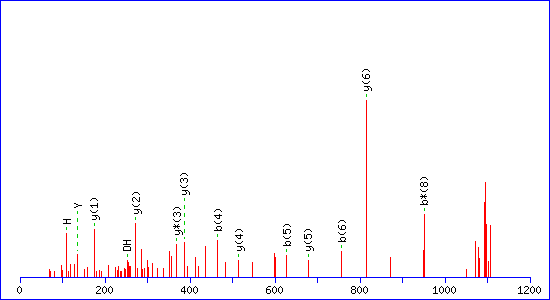


|  | **Immon.** | **a** | **a*** | **a^0^** | **b** | **b*** | **b^0^** | **Seq.** | **v** | **w** | **w'** | **y** | **y*** | **y^0^** | **#** |
| --- | --- | --- | --- | --- | --- | --- | --- | --- | --- | --- | --- | --- | --- | --- | --- |
| **1** | 72.0808 | 72.0808 |  |  | 100.0757 |  |  | **V** |  |  |  |  |  |  | **9** |
| **2** | 86.0964 | 185.1648 |  |  | 213.1598 |  |  | **I** | 985.4122 | 998.4326 | 1012.4483 | 1043.4905 | 1026.4639 | 1025.4799 | **8** |
| **3** | 88.0393 | 300.1918 |  | 282.1812 | 328.1867 |  | 310.1761 | **D** | 870.3853 | 869.3900 |  | 930.4064 | 913.3799 | 912.3959 | **7** |
| **4** | **110.0713** | 437.2507 |  | 419.2401 | **465.2456** |  | 447.2350 | **H** | 733.3264 |  |  | **815.3795** | 798.3529 | 797.3689 | **6** |
| **5** | **136.0757** | 600.3140 |  | 582.3035 | **628.3089** |  | 610.2984 | **Y** | 570.2631 |  |  | **678.3206** | 661.2940 | 660.3100 | **5** |
| **6** | 102.0550 | 729.3566 |  | 711.3461 | **757.3515** |  | 739.3410 | **E** | 441.2205 | 440.2252 |  | **515.2572** | 498.2307 | 497.2467 | **4** |
| **7** | 87.0553 | 843.3995 | 826.3730 | 825.3890 | 871.3945 | 854.3679 | 853.3839 | **N** | 327.1775 | 326.1823 |  | **386.2146** | **369.1881** |  | **3** |
| **8** | 70.0651 | 940.4523 | 923.4258 | 922.4417 | 968.4472 | **951.4207** | 950.4367 | **P** | 230.1248 | 229.1295 |  | **272.1717** | 255.1452 |  | **2** |
| **9** | 129.1135 |  |  |  |  |  |  | **R** | 74.0237 | 73.0284 |  | **175.1190** | 158.0924 |  | **1** |

BIO36833

DWADYLFR 44


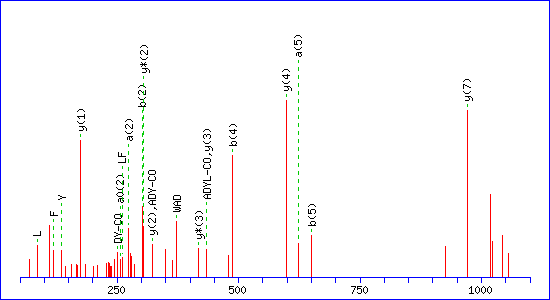


| **#** | **Immon.** | **a** | **a^0^** | **b** | **b^0^** | **Seq.** | **v** | **w** | **y** | **y*** | **y^0^** | **#** |
| --- | --- | --- | --- | --- | --- | --- | --- | --- | --- | --- | --- | --- |
| **1** | 88.0393 | 88.0393 | 70.0287 | 116.0342 | 98.0237 | **D** |  |  |  |  |  | **8** |
| **2** | 159.0917 | **274.1186** | **256.1081** | **302.1135** | 284.1030 | **W** | 839.4046 |  | **970.4781** | 953.4516 | 952.4676 | **7** |
| **3** | 44.0495 | 345.1557 | 327.1452 | **373.1506** | 355.1401 | **A** | 768.3675 |  | 784.3988 | 767.3723 | 766.3883 | **6** |
| **4** | 88.0393 | 460.1827 | 442.1721 | **488.1776** | 470.1670 | **D** | 653.3406 | 652.3453 | 713.3617 | 696.3352 | 695.3511 | **5** |
| **5** | **136.0757** | **623.2460** | 605.2354 | **651.2409** | 633.2304 | **Y** | 490.2772 |  | **598.3348** | 581.3082 |  | **4** |
| **6** | **86.0964** | 736.3301 | 718.3195 | 764.3250 | 746.3144 | **L** | 377.1932 | 376.1979 | **435.2714** | **418.2449** |  | **3** |
| **7** | **120.0808** | 883.3985 | 865.3879 | 911.3934 | 893.3828 | **F** | 230.1248 |  | **322.1874** | **305.1608** |  | **2** |
| **8** | 129.1135 |  |  |  |  | **R** | 74.0237 | 73.0284 | **175.1190** | 158.0924 |  | **1** |

BIO37478

MLPDDINLWYVR 43


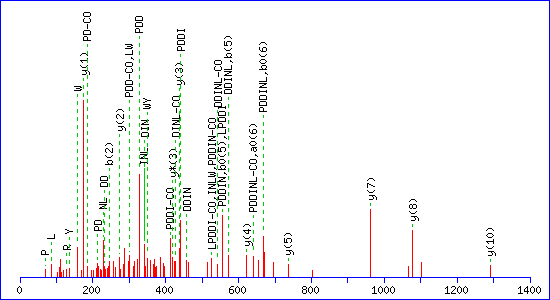


| **#** | **Immon.** | **a** | **a^0^** | **b** | **b^0^** | **Seq.** | **v** | **w** | **y** | **y*** | **y^0^** | **#** |
| --- | --- | --- | --- | --- | --- | --- | --- | --- | --- | --- | --- | --- |
| **1** | 104.0528 | 104.0528 |  | 132.0478 |  | **M** |  |  |  |  |  | **12** |
| **2** | **86.0964** | 217.1369 |  | **245.1318** |  | **L** | 1345.6535 | 1344.6583 | 1403.7318 | 1386.7052 | 1385.7212 | **11** |
| **3** | **70.0651** | 314.1897 |  | 342.1846 |  | **P** | 1248.6008 | 1247.6055 | **1290.6477** | 1273.6212 | 1272.6371 | **10** |
| **4** | 88.0393 | 429.2166 | 411.2061 | 457.2115 | 439.2010 | **D** | 1133.5738 | 1132.5786 | 1193.5949 | 1176.5684 | 1175.5844 | **9** |
| **5** | 88.0393 | 544.2436 | 526.2330 | **572.2385** | **554.2279** | **D** | 1018.5469 | 1017.5516 | **1078.5680** | 1061.5415 | 1060.5574 | **8** |
| **6** | **86.0964** | 657.3276 | **639.3171** | 685.3225 | **667.3120** | **I** | 905.4628 | 918.4832 | **963.5411** | 946.5145 |  | **7** |
| **7** | 87.0553 | 771.3706 | 753.3600 | 799.3655 | 781.3549 | **N** | 791.4199 | 790.4246 | 850.4570 | 833.4305 |  | **6** |
| **8** | **86.0964** | 884.4546 | 866.4441 | 912.4495 | 894.4390 | **L** | 678.3358 | 677.3406 | **736.4141** | 719.3875 |  | **5** |
| **9** | **159.0917** | 1070.5339 | 1052.5234 | 1098.5288 | 1080.5183 | **W** | 492.2565 |  | **623.3300** | 606.3035 |  | **4** |
| **10** | **136.0757** | 1233.5973 | 1215.5867 | 1261.5922 | 1243.5816 | **Y** | 329.1932 |  | **437.2507** | **420.2241** |  | **3** |
| **11** | 72.0808 | 1332.6657 | 1314.6551 | 1360.6606 | 1342.6500 | **V** | 230.1248 | 243.1452 | **274.1874** | 257.1608 |  | **2** |
| **12** | **129.1135** |  |  |  |  | **R** | 74.0237 | 73.0284 | **175.1190** | 158.0924 |  | **1** |

BIO48527

QSLTVLHLIK 38


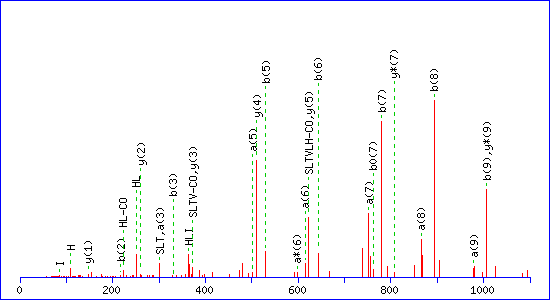


| **#** | **Immon.** | **a** | **a*** | **a^0^** | **b** | **b*** | **b^0^** | **Seq.** | **y** | **y*** | **y^0^** | **#** |
| --- | --- | --- | --- | --- | --- | --- | --- | --- | --- | --- | --- | --- |
| **1** | 102.0549 | 102.0549 | 85.0284 |  | 130.0499 | 113.0233 |  | **Q** |  |  |  | **10** |
| **2** | 60.0444 | 189.0870 | 172.0604 | 171.0764 | **217.0819** | 200.0553 | 199.0713 | **S** | 1023.6561 | **1006.6295** | 1005.6455 | **9** |
| **3** | **86.0964** | **302.1710** | 285.1445 | 284.1605 | **330.1660** | 313.1394 | 312.1554 | **L** | 936.6241 | 919.5975 | 918.6135 | **8** |
| **4** | 74.0600 | 403.2187 | 386.1922 | 385.2082 | 431.2136 | 414.1871 | 413.2031 | **T** | 823.5400 | **806.5135** | 805.5294 | **7** |
| **5** | 72.0808 | **502.2871** | 485.2606 | 484.2766 | **530.2820** | 513.2555 | 512.2715 | **V** | 722.4923 | 705.4658 |  | **6** |
| **6** | **86.0964** | **615.3712** | **598.3446** | 597.3606 | **643.3661** | 626.3396 | 625.3555 | **L** | **623.4239** | 606.3974 |  | **5** |
| **7** | **110.0713** | **752.4301** | 735.4036 | 734.4195 | **780.4250** | 763.3985 | **762.4145** | **H** | **510.3398** | 493.3133 |  | **4** |
| **8** | **86.0964** | **865.5142** | 848.4876 | 847.5036 | **893.5091** | 876.4825 | 875.4985 | **L** | **373.2809** | 356.2544 |  | **3** |
| **9** | **86.0964** | **978.5982** | 961.5717 | 960.5877 | **1006.5932** | 989.5666 | 988.5826 | **I** | **260.1969** | 243.1703 |  | **2** |
| **10** | 101.1073 |  |  |  |  |  |  | **K** | **147.1128** | 130.0863 |  | **1** |

BIO74531

HYAAFER 43


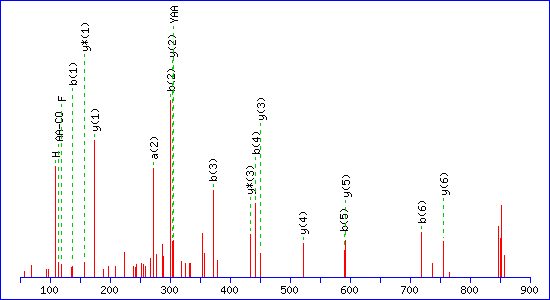


| **#** | **Immon.** | **a** | **b** | **Seq.** | **v** | **y** | **y*** | **y^0^** | **#** |
| --- | --- | --- | --- | --- | --- | --- | --- | --- | --- |
| **1** | **110.0713** | **110.0713** | **138.0662** | **H** |  |  |  |  | **7** |
| **2** | 136.0757 | **273.1346** | **301.1295** | **Y** | 648.3100 | **756.3675** | 739.3410 | 738.3570 | **6** |
| **3** | 44.0495 | 344.1717 | **372.1666** | **A** | 577.2729 | **593.3042** | 576.2776 | 575.2936 | **5** |
| **4** | 44.0495 | 415.2088 | **443.2037** | **A** | 506.2358 | **522.2671** | 505.2405 | 504.2565 | **4** |
| **5** | **120.0808** | 562.2772 | **590.2722** | **F** | 359.1674 | **451.2300** | **434.2034** | 433.2194 | **3** |
| **6** | 102.0550 | 691.3198 | **719.3148** | **E** | 230.1248 | **304.1615** | 287.1350 | 286.1510 | **2** |
| **7** | 129.1135 |  |  | **R** | 74.0237 | **175.1190** | **158.0924** |  | **1** |

BIO25479

LHDVSEEVHR 38


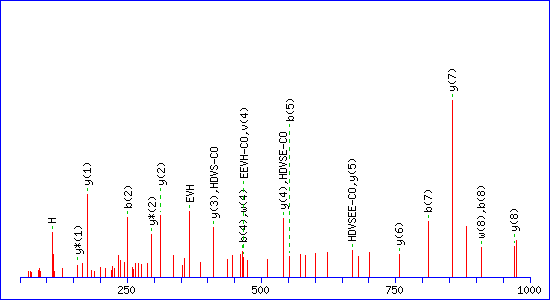


| **#** | **Immon.** | **a** | **a^0^** | **b** | **b^0^** | **Seq.** | **v** | **w** | **y** | **y*** | **y^0^** | **#** |
| --- | --- | --- | --- | --- | --- | --- | --- | --- | --- | --- | --- | --- |
| **1** | 86.0964 | 86.0964 |  | 114.0913 |  | **L** |  |  |  |  |  | **10** |
| **2** | **110.0713** | 223.1553 |  | **251.1503** |  | **H** | 1025.4647 |  | 1107.5178 | 1090.4912 | 1089.5072 | **9** |
| **3** | 88.0393 | 338.1823 | 320.1717 | **366.1772** | 348.1666 | **D** | 910.4377 | **909.4425** | **970.4588** | 953.4323 | 952.4483 | **8** |
| **4** | 72.0808 | 437.2507 | 419.2401 | **465.2456** | 447.2350 | **V** | 811.3693 | 824.3897 | **855.4319** | 838.4054 | 837.4213 | **7** |
| **5** | 60.0444 | 524.2827 | 506.2722 | **552.2776** | 534.2671 | **S** | 724.3373 | 723.3420 | **756.3635** | 739.3369 | 738.3529 | **6** |
| **6** | 102.0550 | 653.3253 | 635.3148 | 681.3202 | 663.3097 | **E** | 595.2947 | 594.2994 | **669.3315** | 652.3049 | 651.3209 | **5** |
| **7** | 102.0550 | 782.3679 | 764.3573 | **810.3628** | 792.3523 | **E** | **466.2521** | **465.2568** | **540.2889** | 523.2623 | 522.2783 | **4** |
| **8** | 72.0808 | 881.4363 | 863.4258 | **909.4312** | 891.4207 | **V** | 367.1837 | 380.2041 | **411.2463** | 394.2197 |  | **3** |
| **9** | **110.0713** | 1018.4952 | 1000.4847 | 1046.4901 | 1028.4796 | **H** | 230.1248 |  | **312.1779** | **295.1513** |  | **2** |
| **10** | 129.1135 |  |  |  |  | **R** | 74.0237 | 73.0284 | **175.1190** | **158.0924** |  | **1** |

B. Novel proteins MS/MS spectra:

BIO68373

RGNPPFQFR 57


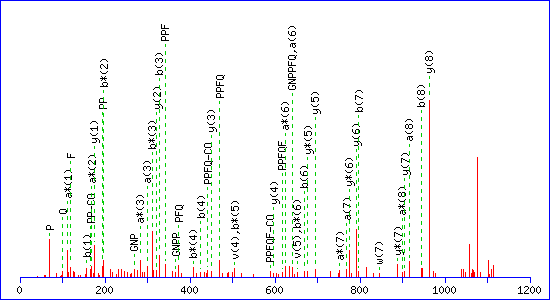


| **#** | **Immon.** | **a** | **a*** | **b** | **b*** | **d** | **Seq.** | **v** | **w** | **y** | **y*** | **#** |
| --- | --- | --- | --- | --- | --- | --- | --- | --- | --- | --- | --- | --- |
| **1** | 129.1135 | 129.1135 | **112.0869** | **157.1084** | 140.0818 | 44.0495 | **R** |  |  |  |  | **9** |
| **2** | 30.0338 | 186.1349 | **169.1084** | 214.1299 | **197.1033** |  | **G** |  |  | **962.4843** | 945.4577 | **8** |
| **3** | 87.0553 | **300.1779** | **283.1513** | **328.1728** | **311.1462** | 257.1721 | **N** | 846.4257 | **845.4305** | **905.4628** | **888.4363** | **7** |
| **4** | **70.0651** | 397.2306 | 380.2041 | **425.2255** | **408.1990** | 371.2150 | **P** | 749.3729 | 748.3777 | **791.4199** | **774.3933** | **6** |
| **5** | **70.0651** | 494.2834 | 477.2568 | 522.2783 | **505.2518** | 468.2677 | **P** | **652.3202** | 651.3249 | **694.3671** | **677.3406** | **5** |
| **6** | **120.0808** | **641.3518** | **624.3253** | **669.3467** | **652.3202** |  | **F** | **505.2518** |  | **597.3144** | 580.2878 | **4** |
| **7** | **101.0709** | **769.4104** | **752.3838** | **797.4053** | 780.3787 | 712.3889 | **Q** | 377.1932 | 376.1979 | **450.2459** | 433.2194 | **3** |
| **8** | **120.0808** | **916.4788** | **899.4522** | **944.4737** | 927.4472 |  | **F** | 230.1248 |  | **322.1874** | 305.1608 | **2** |
| **9** | 129.1135 |  |  |  |  |  | **R** | 74.0237 | 73.0284 | **175.1190** | 158.0924 | **1** |

BIO43803

HLFQIR 37 KEQLFVELVR 40 validated by RT-PCR

HLFQIR 37


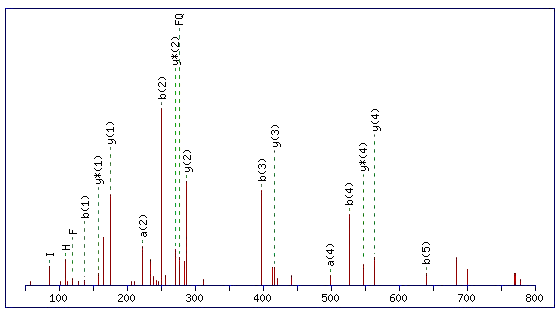


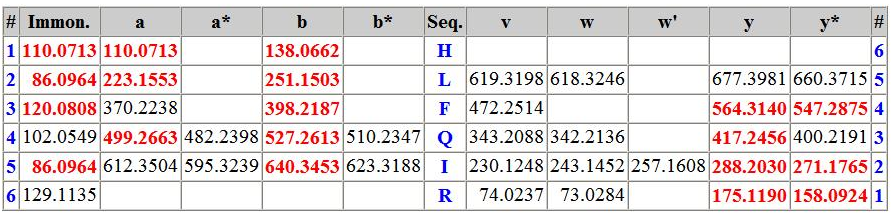


KEQLFVELVR 40


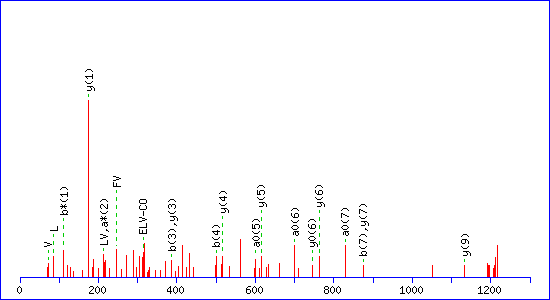


| **#** | **Immon.** | **a** | **a*** | **a^0^** | **b** | **b*** | **b^0^** | **Seq.** | **v** | **w** | **y** | **y*** | **y^0^** | **#** |
| --- | --- | --- | --- | --- | --- | --- | --- | --- | --- | --- | --- | --- | --- | --- |
| **1** | 101.1073 | 101.1073 | 84.0808 |  | 129.1022 | **112.0757** |  | **K** |  |  |  |  |  | **10** |
| **2** | 102.0550 | 230.1499 | **213.1234** | 212.1394 | 258.1448 | 241.1183 | 240.1343 | **E** | 1059.5833 | 1058.5881 | **1133.6201** | 1116.5935 | 1115.6095 | **9** |
| **3** | 102.0549 | 359.1925 | 342.1660 | 341.1819 | **387.1874** | 370.1609 | 369.1769 | **Q** | 930.5407 | 929.5455 | 1004.5775 | 987.5510 | 986.5669 | **8** |
| **4** | **86.0964** | 472.2766 | 455.2500 | 454.2660 | **500.2715** | 483.2449 | 482.2609 | **L** | 817.4567 | 816.4614 | **875.5349** | 858.5084 | 857.5243 | **7** |
| **5** | 120.0808 | 619.3450 | 602.3184 | **601.3344** | 647.3399 | 630.3133 | 629.3293 | **F** | 670.3883 |  | **762.4509** | 745.4243 | **744.4403** | **6** |
| **6** | **72.0808** | 718.4134 | 701.3868 | **700.4028** | 746.4083 | 729.3818 | 728.3977 | **V** | 571.3198 | 584.3402 | **615.3824** | 598.3559 | 597.3719 | **5** |
| **7** | 102.0550 | 847.4560 | 830.4294 | **829.4454** | **875.4509** | 858.4244 | 857.4403 | **E** | 442.2772 | 441.2820 | **516.3140** | 499.2875 | 498.3035 | **4** |
| **8** | **86.0964** | 960.5401 | 943.5135 | 942.5295 | 988.5350 | 971.5084 | 970.5244 | **L** | 329.1932 | 328.1979 | **387.2714** | 370.2449 |  | **3** |
| **9** | **72.0808** | 1059.6085 | 1042.5819 | 1041.5979 | 1087.6034 | 1070.5768 | 1069.5928 | **V** | 230.1248 | 243.1452 | 274.1874 | 257.1608 |  | **2** |
| **10** | 129.1135 |  |  |  |  |  |  | **R** | 74.0237 | 73.0284 | **175.1190** | 158.0924 |  | **1** |

BIO07235

WLNGLLLR 53 validated by RT-PCR

**
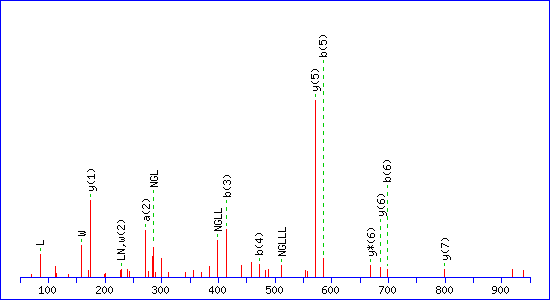
**

| **#** | **Immon.** | **a** | **a*** | **b** | **b*** | **Seq.** | **v** | **w** | **y** | **y*** | **#** |
| --- | --- | --- | --- | --- | --- | --- | --- | --- | --- | --- | --- |
| **1** | **159.0917** | **159.0917** |  | 187.0866 |  | **W** |  |  |  |  | **8** |
| **2** | **86.0964** | **272.1757** |  | 300.1707 |  | **L** | 741.4254 | 740.4301 | **799.5036** | 782.4771 | **7** |
| **3** | 88.0393 | 387.2027 | 370.1761 | **415.1976** | 398.1710 | **N** | 626.3984 | 625.4032 | **686.4195** | **669.3930** | **6** |
| **4** | 30.0338 | 444.2241 | 427.1976 | **472.2191** | 455.1925 | **G** |  |  | **571.3926** | 554.3661 | **5** |
| **5** | **86.0964** | 557.3082 | 540.2817 | **585.3031** | 568.2766 | **L** | 456.2929 | 455.2976 | 514.3711 | 497.3446 | **4** |
| **6** | **86.0964** | 670.3923 | 653.3657 | **698.3872** | 681.3606 | **L** | 343.2088 | 342.2136 | 401.2871 | 384.2605 | **3** |
| **7** | **86.0964** | 783.4763 | 766.4498 | 811.4712 | 794.4447 | **L** | 230.1248 | **229.1295** | 288.2030 | 271.1765 | **2** |
| **8** | 129.1135 |  |  |  |  | **R** | 74.0237 | 73.0284 | **175.1190** | 158.0924 | **1** |

BIO48527

QSLTVLHLIK 38


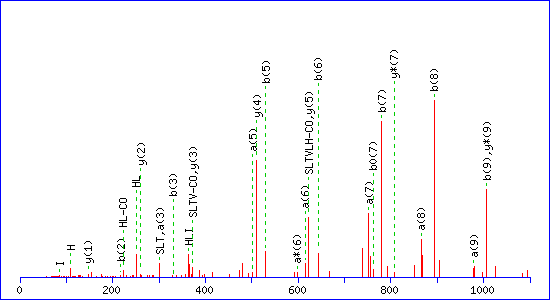


| **#** | **Immon.** | **a** | **a*** | **a^0^** | **b** | **b*** | **b^0^** | **Seq.** | **y** | **y*** | **y^0^** | **#** |
| --- | --- | --- | --- | --- | --- | --- | --- | --- | --- | --- | --- | --- |
| **1** | 102.0549 | 102.0549 | 85.0284 |  | 130.0499 | 113.0233 |  | **Q** |  |  |  | **10** |
| **2** | 60.0444 | 189.0870 | 172.0604 | 171.0764 | **217.0819** | 200.0553 | 199.0713 | **S** | 1023.6561 | **1006.6295** | 1005.6455 | **9** |
| **3** | **86.0964** | **302.1710** | 285.1445 | 284.1605 | **330.1660** | 313.1394 | 312.1554 | **L** | 936.6241 | 919.5975 | 918.6135 | **8** |
| **4** | 74.0600 | 403.2187 | 386.1922 | 385.2082 | 431.2136 | 414.1871 | 413.2031 | **T** | 823.5400 | **806.5135** | 805.5294 | **7** |
| **5** | 72.0808 | **502.2871** | 485.2606 | 484.2766 | **530.2820** | 513.2555 | 512.2715 | **V** | 722.4923 | 705.4658 |  | **6** |
| **6** | **86.0964** | **615.3712** | **598.3446** | 597.3606 | **643.3661** | 626.3396 | 625.3555 | **L** | **623.4239** | 606.3974 |  | **5** |
| **7** | **110.0713** | **752.4301** | 735.4036 | 734.4195 | **780.4250** | 763.3985 | **762.4145** | **H** | **510.3398** | 493.3133 |  | **4** |
| **8** | **86.0964** | **865.5142** | 848.4876 | 847.5036 | **893.5091** | 876.4825 | 875.4985 | **L** | **373.2809** | 356.2544 |  | **3** |
| **9** | **86.0964** | **978.5982** | 961.5717 | 960.5877 | **1006.5932** | 989.5666 | 988.5826 | **I** | **260.1969** | 243.1703 |  | **2** |
| **10** | 101.1073 |  |  |  |  |  |  | **K** | **147.1128** | 130.0863 |  | **1** |

BIO58539

MKVIIISR 54

**
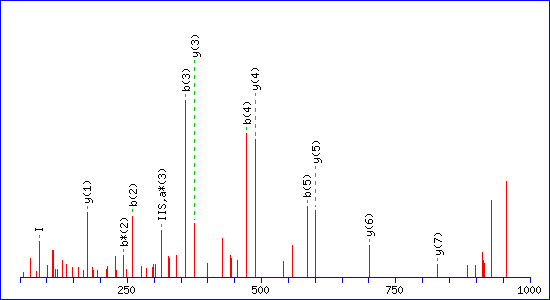
**

| **#** | **Immon.** | **a** | **a*** | **b** | **b*** | **Seq.** | **v** | **w** | **y** | **y*** | **y^0^** | **#** |
| --- | --- | --- | --- | --- | --- | --- | --- | --- | --- | --- | --- | --- |
| **1** | 104.0528 | 104.0528 |  | 132.0478 |  | **M** |  |  |  |  |  | **8** |
| **2** | 101.1073 | 232.1478 | 215.1213 | **260.1427** | **243.1162** | **K** | 755.4774 | 754.4822 | **828.5665** | 811.5400 | 810.5560 | **7** |
| **3** | 72.0808 | 331.2162 | **314.1897** | **359.2111** | 342.1846 | **V** | 656.4090 | 669.4294 | **700.4716** | 683.4450 | 682.4610 | **6** |
| **4** | **86.0964** | 444.3003 | 427.2737 | **472.2952** | 455.2687 | **I** | 543.3249 | 556.3453 | **601.4032** | 584.3766 | 583.3926 | **5** |
| **5** | **86.0964** | 557.3844 | 540.3578 | **585.3793** | 568.3527 | **I** | 430.2409 | 443.2613 | **488.3191** | 471.2926 | 470.3085 | **4** |
| **6** | **86.0964** | 670.4684 | 653.4419 | 698.4633 | 681.4368 | **I** | 317.1568 | 330.1772 | **375.2350** | 358.2085 | 357.2245 | **3** |
| **7** | 60.0444 | 757.5004 | 740.4739 | 785.4954 | 768.4688 | **S** | 230.1248 | 229.1295 | 262.1510 | 245.1244 | 244.1404 | **2** |
| **8** | 129.1135 |  |  |  |  | **R** | 74.0237 | 73.0284 | **175.1190** | 158.0924 |  | **1** |

BIO01608

DIINVLLR 68 validated by RT-PCR

**
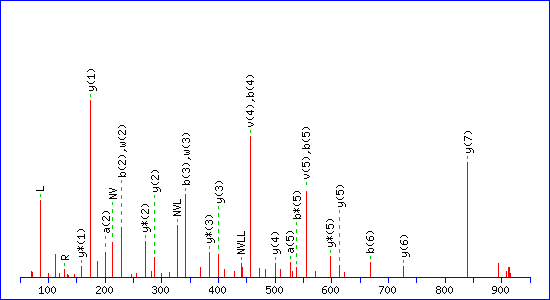
**

| **#** | **Immon.** | **a** | **a^0^** | **b** | **b*** | **b^0^** | **Seq.** | **v** | **w** | **y** | **y*** | **#** |
| --- | --- | --- | --- | --- | --- | --- | --- | --- | --- | --- | --- | --- |
| **1** | 88.0393 | 88.0393 | 70.0287 | 116.0342 |  | 98.0237 | **D** |  |  |  |  | **8** |
| **2** | **86.0964** | **201.1234** | 183.1128 | **229.1183** |  | 211.1077 | **I** | 782.4883 | 795.5087 | **840.5665** | 823.5400 | **7** |
| **3** | **86.0964** | 314.2074 | 296.1969 | **342.2023** |  | 324.1918 | **I** | 669.4042 | 682.4246 | **727.4825** | 710.4559 | **6** |
| **4** | 87.0553 | 428.2504 | 410.2398 | **456.2453** | 439.2187 | 438.2347 | **N** | **555.3613** | 554.3661 | **614.3984** | **597.3719** | **5** |
| **5** | 72.0808 | **527.3188** | 509.3082 | **555.3137** | **538.2871** | 537.3031 | **V** | **456.2929** | 469.3133 | **500.3555** | 483.3289 | **4** |
| **6** | **86.0964** | 640.4028 | 622.3923 | **668.3978** | 651.3712 | 650.3872 | **L** | 343.2088 | **342.2136** | **401.2871** | **384.2605** | **3** |
| **7** | **86.0964** | 753.4869 | 735.4763 | 781.4818 | 764.4553 | 763.4713 | **L** | 230.1248 | **229.1295** | **288.2030** | **271.1765** | **2** |
| **8** | **129.1135** |  |  |  |  |  | **R** | 74.0237 | 73.0284 | **175.1190** | **158.0924** | **1** |

BIO50043

IIAGLEHQTSGHIR 62 validated by RT-PCR

**
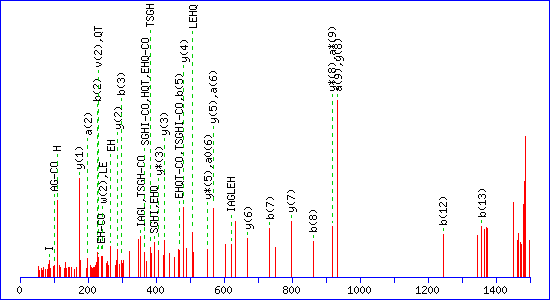
**

| **#** | **Immon.** | **a** | **a^0^** | **b** | **b*** | **b^0^** | **Seq.** | **v** | **w** | **y** | **y*** | **#** |
| --- | --- | --- | --- | --- | --- | --- | --- | --- | --- | --- | --- | --- |
| **1** | **86.0964** | **86.0964** |  | 114.0913 |  |  | **I** |  |  |  |  | **14** |
| **2** | **86.0964** | **199.1805** |  | **227.1754** |  |  | **I** | 1360.6716 | 1373.6920 | 1418.7499 | 1401.7233 | **13** |
| **3** | 44.0495 | 270.2176 |  | **298.2125** |  |  | **A** | 1289.6345 |  | 1305.6658 | 1288.6393 | **12** |
| **4** | 30.0338 | 327.2391 |  | **355.2340** |  |  | **G** |  |  | 1234.6287 | 1217.6022 | **11** |
| **5** | **86.0964** | 440.3231 |  | **468.3180** |  |  | **L** | 1119.5290 | 1118.5337 | 1177.6072 | 1160.5807 | **10** |
| **6** | 102.0550 | **569.3657** | **551.3552** | 597.3606 |  | 579.3501 | **E** | 990.4864 | 989.4912 | 1064.5232 | 1047.4966 | **9** |
| **7** | **110.0713** | 706.4246 | 688.4141 | **734.4196** |  | 716.4090 | **H** | 853.4275 |  | **935.4806** | **918.4540** | **8** |
| **8** | **101.0709** | 834.4832 | 816.4726 | **862.4781** | 845.4516 | 844.4676 | **Q** | 725.3689 | 724.3737 | **798.4217** | 781.3951 | **7** |
| **9** | 74.0600 | **935.5309** | 917.5203 | 963.5258 | 946.4993 | 945.5152 | **T** | 624.3212 | 637.3416 | **670.3631** | 653.3365 | **6** |
| **10** | 60.0444 | 1022.5629 | 1004.5524 | 1050.5578 | 1033.5313 | 1032.5473 | **S** | 537.2892 | 536.2940 | **569.3154** | **552.2889** | **5** |
| **11** | 30.0338 | 1079.5844 | 1061.5738 | 1107.5793 | 1090.5528 | 1089.5687 | **G** |  |  | **482.2834** | 465.2568 | **4** |
| **12** | **110.0713** | 1216.6433 | 1198.6327 | **1244.6382** | 1227.6117 | 1226.6276 | **H** | 343.2088 |  | **425.2619** | **408.2354** | **3** |
| **13** | **86.0964** | 1329.7274 | 1311.7168 | **1357.7223** | 1340.6957 | 1339.7117 | **I** | **230.1248** | **243.1452** | **288.2030** | 271.1765 | **2** |
| **14** | 129.1135 |  |  |  |  |  | **R** | 74.0237 | 73.0284 | **175.1190** | 158.0924 | **1** |
