# Supplementary material for: A proteogenomic analysis of Shigella flexneri using 2D LC-MALDI TOF/TOF
Source: BMC Genomics. 2011 Oct 28;12:528. doi: 10.1186/1471-2164-12-528 (PMC3219829; doi:10.1186/1471-2164-12-528)
Supplement: Additional file 5 — Supplementary Table S3. Table S3 shows a list of primers used in this article. [file 1471-2164-12-528-S5.DOC]

**Table S3.** **Primers used in this article**

| **Primer name** | **Primer sequence（from 5′ to 3′ ）** | **Product size（bp）** |
| --- | --- | --- |
| *yhdP*(F) | TGGCTCGGTAAATGGATGC | 506 |
| *yhdP*(R) | GCCTGAAAACGGGTCTTGT |
| *yebJ*(F) | CGCAATTACGATCCGCTTTA | 474 |
| *yebJ*(R) | CGCCGTCTTTGGTGATTTCTA |
| *smpA*(F) | AGCGAGTGGTTTACCGTCCTG | 199 |
| *smpA*(R) | GTTTGCTGCGTTACACCTTCAT |
| *zwf*(F) | GAAGTGGGGATCGAAGGG | 368 |
| *zwf*(R) | CAGTACGCAGGTAGAATGGC |
| *fus*A(F) | ATCCCTGGCGAATACATCC | 376 |
| *fusA*(R) | GCTGAGTTGCGTATCCGA |
| BIO43803(F) | GCCGCGCTTGTTGAAAAC | 140 |
| BIO43803(R) | CGCACAATCGCGAGCAAC |
| BIO01608(F) | TGAACCCAATATCTTTCCTTAGC | 118 |
| BIO01608(R) | AACGACCAACTCCAGAGCACA |
| BIO50043(F) | GGGCATATTCGCTTCCACG | 444 |
| BIO50043(R) | GGCTACTTCGGTCGCCTCTT |
| BIO07235(F) | ATGTTCGGGTCGCAGCATCG | 78 |
| BIO07235 (R) | TTATTGTTCCTTCCTACGCAAC |
| *ipaD*(F) | CCTTACTATGCTCAACGACACC | 588 |
| *ipaD*(R) | TGAGATACCTTGCCGATTGTTC |
| negative control(F) | ACCGAGGAATGAATAAAGAA | - |
| negative control(R) | TACGAAAACCAGTATTAACCAC |
| BIO11778(F1) | GAATGCCACAACGACACCAC | 369 |
| BIO11778(R1) | CCGGGATTTCATCCACCTTA |
| BIO11778(F2) | GACAGAAACTCGTCCACTG | 246 |
| BIO11778(R2) | AACGTGACAGATCGGTTG |
